# Inhibition of autophagy ameliorates pulmonary microvascular dilation and PMVECs excessive proliferation in rat experimental hepatopulmonary syndrome

**DOI:** 10.1038/srep30833

**Published:** 2016-08-02

**Authors:** Duo Xu, Bing Chen, Jianteng Gu, Lin Chen, Karine Belguise, Xiaobo Wang, Bin Yi, Kaizhi Lu

**Affiliations:** 1 Department of Anesthesia, Southwest Hospital, Third Military Medical University, Chongqing 400038, China; 2 University P. Sabatier Toulouse 3 and CNRS, LBCMCP, 31062 Toulouse Cedex 9, France

## Abstract

Hepatopulmonary syndrome (HPS) is a defective liver-induced pulmonary vascular disorder with massive pulmonary microvascular dilation and excessive proliferation of pulmonary microvascular endothelial cells (PMVECs). Growing evidence suggests that autophagy is involved in pulmonary diseases, protectively or detrimentally. Thus, it is interesting and important to explore whether autophagy might be involved in and critical in HPS. In the present study, we report that autophagy was activated in common bile duct ligation (CBDL) rats and cultured pulmonary PMVECs induced by CBDL rat serum, two accepted *in vivo* and *in vitro* experimental models of HPS. Furthermore, pharmacological inhibition of autophagy with 3-methyladenine (3-MA) significantly alleviated pathological alterations and typical symptom of HPS in CBDL rats *in vivo*, and consistently 3-MA significantly attenuated the CBDL rat serum-induced excessive proliferation of PMVECs *in vitro*. All these changes mediated by 3-MA might explain the observed prominent improvement of pulmonary appearance, edema, microvascular dilatation and arterial oxygenation *in vivo*. Collectively, these results suggest that autophagy activation may play a critical role in the pathogenesis of HPS, and autophagy inhibition may have a therapeutic potential for this disease.

Hepatopulmonary syndrome (HPS) is a life-threatening disease characterized by a triad of chronic liver disease (CLD), intrapulmonary vascular dilation (IPVD) and serious hypoxemia^1^,^2^. The prevalence of HPS varies from 4–47% due to different cut-offs in defining arterial hypoxemia in primary studies, and its mortality rate is about 41%^3^,^4^. Although progress has been made in delineating the mechanisms underlying the imbalance of vasoactive substances, pulmonary vascular alterations and angiogenesis in HPS, to date, there is still lack of effective therapeutic approaches apart from liver transplantation (LT)^5^,^6^,^7^.

Autophagy (derived from the Greek words meaning “self eating”) is identified as an evolutionarily conserved cellular housekeeping process that is involved in the degradation of protein and organelle^8^,^9^. Increasing evidence has demonstrated that the dysfunction of autophagy contributes to various diseases, such as cancer, atherosclerosis, Alzheimer’s disease and acute lung injury (ALI)^10^,^11^,^12^,^13^. In certain circumstances, autophagy plays a protective role via its clearing the damaged and unhealthy organelles. For example, autophagy is involved in stress adaption in lung injury through its removal of the damaged organelles and thus the promotion of cell survival^14^. However, the massive and persistent activation of autophagy may contribute to excessive cell proliferation and pathological angiogenesis^15^,^16^. HPS is a kind of pulmonary vascular complication with high mortality rate^17^. Considering that autophagy is involved in and critical in lung injury, protectively or detrimentally. Thus, it is interesting and important to explore whether autophagy might be involved in and critical in HPS. However, it is still unclear how the process of autophagy is altered in the pathogenesis of HPS and how this alteration is detrimental or beneficial to HPS.

At the cellular level, our previous research demonstrated that the common bile duct ligation (CBDL) rat serum induces the excessive proliferation of pulmonary microvascular endothelial cells (PMVECs) *in vitro*, which could contribute to the HPS-associated angiogenesis, a highly complicated and regulated process to form new vessels and capillary networks^18^,^19^,^20^. In the present study, we hypothesized that the initial autophagy activation may play a pivotal role in the pathological alterations of HPS. To address this hypothesis, we assessed whether autophagy level is increased in both CBDL rats and cultured PMVECs under the stimulation of CBDL rat serum, and whether autophagy inhibition could affect the pathological status of HPS in both *in vivo* and *in vitro* models.

## Results

### Activation of autophagy in lung tissues of CBDL rats

**Figure 1:**
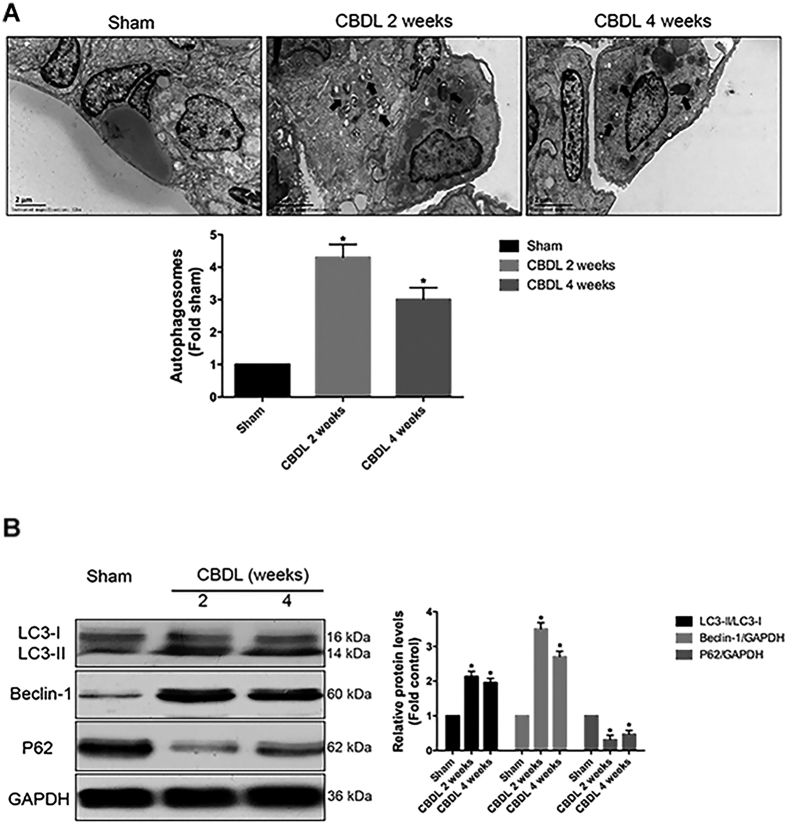
Activation of autophagy in lung tissues after common bile duct ligation (CBDL) *in vivo*. (**A**) Representative transmission electron microscopy (TEM) images and graphical summaries of autophagosomes in lung tissues of sham, 2- and 4-week CBDL rats (n = 5). Broad arrows represent autophagosomes. 10 fields for each rat were observed. (**B**) Western blotting and graphical summaries of LC3B, Beclin-1 and p62 protein levels in lung tissues of sham, 2- and 4-week CBDL rats (n = 5). All blots were representative of three independent experiments. Values were expressed as means ± SEM. *P < 0.05 compared with sham.

Autophagy status is defined by the presence and number of autophagosomes within cells. Thus, we assessed autophagy status by the detection of autophagosomes in lung tissues of CBDL rats with transmission electron microscopy (TEM). Autophagosomes, which are the double membrane structures or double membrane vacuoles, were prominently observed in lung tissues of 2- and 4-week CBDL rats (Fig. 1A, as indicated by broad arrows), while they were weakly detectable in the control sham rats. The most abundant autophagosomes were observed in 2-week CBDL rats, and there is a slightly decrease in 4-week CBDL rats. Next, we determined the expression levels of autophagy-related proteins (LC3B, Beclin-1 and P62) in lung tissues of CBDL rats, as evaluated with Western blotting. LC3B is required for the formation of autophgosome membranes, and upon induction of autophagy cytosolic LC3-I is cleaved and lipidated to form LC3-II. Beclin-1 forms a protein complex with the Class III PI3K, which is critical for the recruitment of LC3B. p62 degradation reflects the lysozyme fusion and autophagosome breakdown. So autophagy activation is commonly evidenced by the increase in LC3-II/LC3-I and Beclin-1 protein levels, and reduction in p62 protein levels^8^. Our results demonstrated that the protein levels of LC3B and Beclin-1 in the lung tissues were significantly increased and peaked at 2-week CBDL rats; oppositely, the protein levels of p62 in lung tissues were significantly decreased and dropped to minimal in 2-week CBDL rats; the positive or negative changes of all these proteins showed a slightly reduction in 4-week CBDL rats, and all these temporal changes are consistent with our observation of autophagosome formation (Fig. 1B). Both the formation of autophagosomes and the respective changes of autophagy-related proteins demonstrated the activation of autophagy in lung tissues of CBDL rats.

### Activation of autophagy in cultured PMVECs under the stimulation of CBDL rat serum

**Figure 2:**
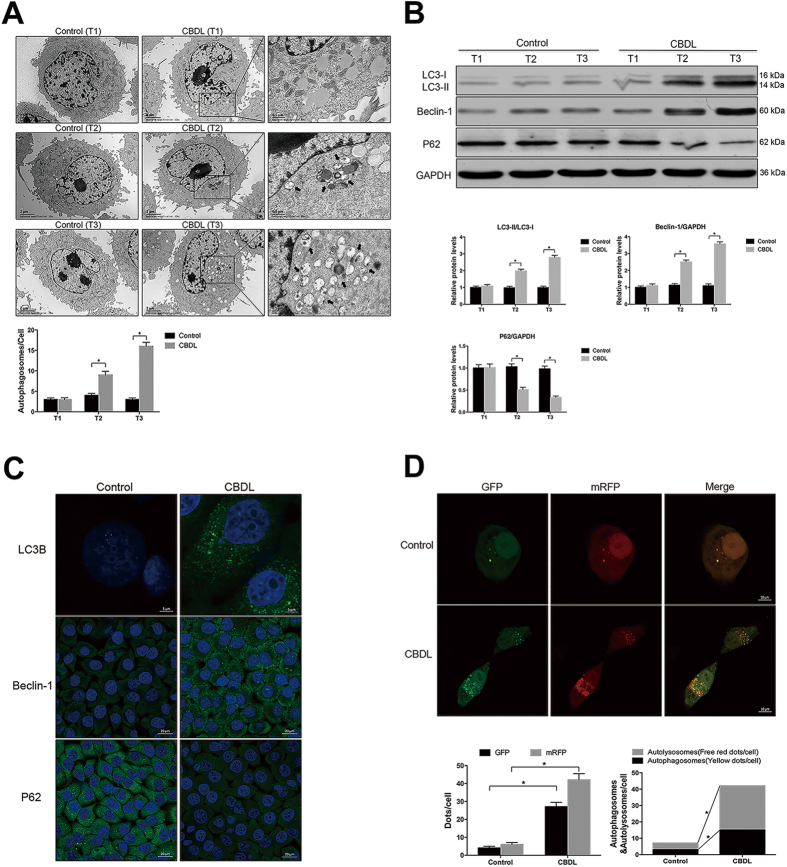
Activation of autophagy in cultured PMVECs under the stimulation of CBDL rat serum *in vitro*. (**A**) Representative TEM images and graphical summaries of autophagosomes in cultured PMVECs under the stimulation of normal rat serum or CBDL rat serum for 0 h (T1), 12 h (T2) and 24 h (T3). Broad arrows represent autophgosomes. Asterisks represent autolysosomes. 30 random cells for each group were observed. (**B**) Western blotting and graphical summaries of LC3B, Beclin-1 and p62 protein levels in cultured PMVECs under the stimulation of normal rat serum or CBDL rat serum for 0 h (T1), 12 h (T2) and 24 h (T3). All blots were representative of three independent experiments. (**C**) Representative immunocytochemistry images of LC3B, Beclin-1 and p62 protein in cultured PMVECs under the stimulation of normal rat serum or CBDL rat serum for 24 h. (**D**) Representative confocal microscope images and graphical summaries of LC3 in different groups of PMVECs infected with mRFP-GFP-LC3 adenovirus for 24 h. 30 random cells for each group were observed. Values were expressed as means ± SEM. *P < 0.05.

Because PMVECs is a major cell type in pulmonary microvasculature and our previous research has demonstrated that the excessive proliferation of PMVECs contributes to the development of HPS^20^,^21^. Thus, in this part we asked whether autophagy activation observed *in vivo* could be also detected in the *in vitro* cultured cell model. The formation of autophagosomes in cultured PMVECs, under the stimulation of normal rat serum or CBDL rat serum for 0 h (T1), 12 h (T2) and 24 h (T3), was detected with TEM. The control PMVECs contained normal organelles, nucleus and chromatin. After the stimulation with CBDL serum for different time points, PMVECs were observed to contain a number of typical autophagosomes in the cytoplasm (Fig. 2A, as indicated by broad arrows). The most abundant autophagosomes were observed at 24 h after the stimulation of CBDL rat serum. In addition to the typical autophagosomes with engulfed organelles, the fusion of autophagosomes with lysosomes was occasionally detected in PMVECs after the CBDL rat serum stimulation (Fig. 2A, as indicated by asterisk). The expression levels of autophagy-related proteins (LC3B, Beclin-1 and P62) in cultured PMVECs, under the stimulation of normal rat serum or CBDL rat serum, were evaluated with Western blotting and immunocytochemistry. Our results demonstrated that the protein levels of LC3B and Beclin-1 in cultured PMVECs were significantly increased and peaked at 24 h after the CBDL rat serum stimulation, while the protein levels of p62 were significantly decreased and dropped to minimal at 24 h after the CBDL rat serum stimulation (Fig. 2B,C).

Since autophagy is a dynamic cellular process, we analyzed the autophagic flux status of PMVECs under the stimulation of normal rat serum or CBDL rat serum for 24 h, as evidenced with the spatial distribution of mCherry red fluorescent protein (mRFP) and green fluorescent protein (GFP) from the expression of mRFP-GFP-LC3 adenovirus (Fig. 2D). Based on the pH sensitivity difference between GFP and mRFP, neutral autophagosomes and acidic autolysosomes are labeled with yellow color or red color in merged images respectively^22^,^23^. The numbers of GFP and mRFP dots in PMVECs were significantly increased after CBDL rat serum stimulation. In the merged images, both numbers of yellow dots and free red dots were increased, indicating that the accumulation of both autophagosomes (yellow puncta) and autolysosomes (red puncta) was induced in PMVECs after CBDL rat serum stimulation. These findings further demonstrated that CBDL rat serum could induce autophagy activation in cultured PMVECs. Taken together, we can conclude that autophagy is activated in both *in vivo* and *in vitro* models of HPS.

### Inhibition of autophagy improved pulmonary appearance, histology, microvascular dilatation and arterial oxygenation in CBDL rats

**Figure 3:**
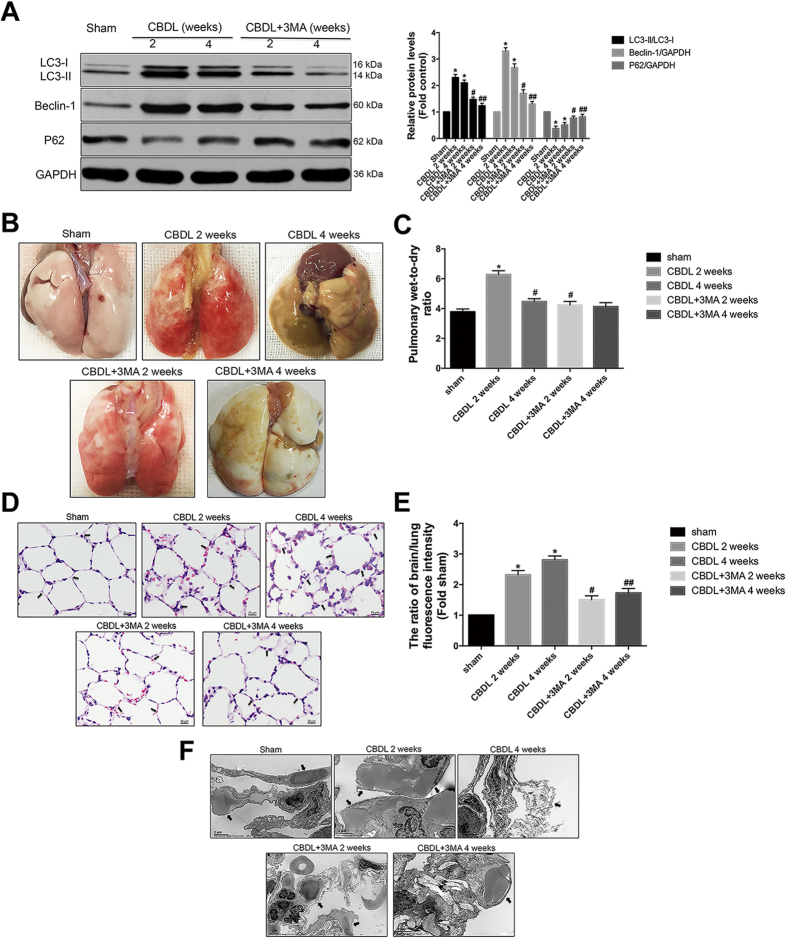
Inhibition of autophagy with 3-methyladenien (3MA)-15 mg/kg improved pulmonary appearance, histology and microvascular dilatation after CBDL *in vivo*. (**A**) Western blotting and graphical summaries of LC3B, Beclin-1 and p62 protein levels in lung tissues of sham, 2- and 4-week CBDL rats with or without 3MA administration (n = 5). All blots were representative of three independent experiments. (**B**) Representative pictures of lung tissue samples in sham, 2- and 4-week CBDL rats with or without 3MA administration. 3MA alleviated pulmonary hemorrhage and old petechial in 2- and 4-week CBDL rats. (**C**) Graphical summaries of pulmonary wet-to-dry ratio in sham, 2- and 4-week CBDL rats with or without 3MA administration (n = 5). 3MA improved pulmonary edema in 2-week CBDL rats, as evidenced by the decreased pulmonary wet-to-dry ratio. The ratio in the 4-week CBDL rats decreased to the baseline, and there is no significant difference between the 4-week CBDL rats with or without 3MA administration. (**D**) Representative micrographs of haematoxylin and eosin staining of pulmonary microvessels (indicated by arrows) in sham, 2- and 4-week CBDL rats with or without 3MA administration. 3MA alleviated the disorganized and enlarged microvessels in 2- and 4-week CBDL rats. (**E**) Graphical summaries of fluorescent-labeled microsphere assay in sham, 2- and 4-week CBDL rats with or without 3MA administration (n = 5). 3MA improved pulmonary microvasular dilation in 2- and 4-week CBDL rats, as evidenced by the decreased ratio of brain-over-lung. (**F**) Representative TEM images of lung tissues in sham, 2- and 4-week CBDL rats with or without 3MA administration. 2- and 4-week CBDL rats had enlarged microvessels filled with erythrocytes and destructive pulmonary microvasculature with exfoliated alveolar epithelium, respectively (indicated by arrows). 3MA significantly alleviated the above alterations. Values were expressed as means ± SEM. *P < 0.05 compared with sham. ^#^*P* < 0.05 compared with CBDL 2-week. ^##^*P* < 0.05 compared with CBDL 4-week.

The main question is whether activation of autophagy might play an important role in the pathology of HPS. Thus, we evaluated whether autophagy inhibition influenced the pathological alterations and typical symptom of HPS. Firstly, we confirmed the inhibitory effect of 3-methyladenien (3MA), the widely used inhibitor of autophagy. 3MA(15 mg/kg) effectively blocked autophagy activation in CBDL rats, as evidenced by the decreased protein levels of LC3B and Beclin-1 as well as the increased protein levels of p62 (Fig. 3A). The pathological alterations of HPS are evaluated by pulmonary appearance, histology and microvascular dilatation^24^. Therefore, we next assessed the effect of autophagy inhibition on all these 3 standards with typical lung tissue samples, pulmonary wet-to-dry ratio, haematoxylin and eosin (HE) staining, fluorescent-labeled microsphere assay and TEM. Compared with the sham group, 2- and 4-week CBDL rats had serious pulmonary hemorrhage, old petechial, edema and microvasular injuries, while 3MA administration significantly alleviated these histological alterations (Fig. 3B–F). Similarly, arterial blood gas analysis demonstrated that both 2- and 4-week CBDL rats had the decreased levels of *PaO*_*2*_ and the increased levels of *A*_*a*_*PO*, while 3MA treatment strongly improved pulmonary oxygenation function (Table 1).

### Inhibition of autophagy attenuated the excessive proliferation of PMVECs induced by CBDL rat serum

**Figure 4:**
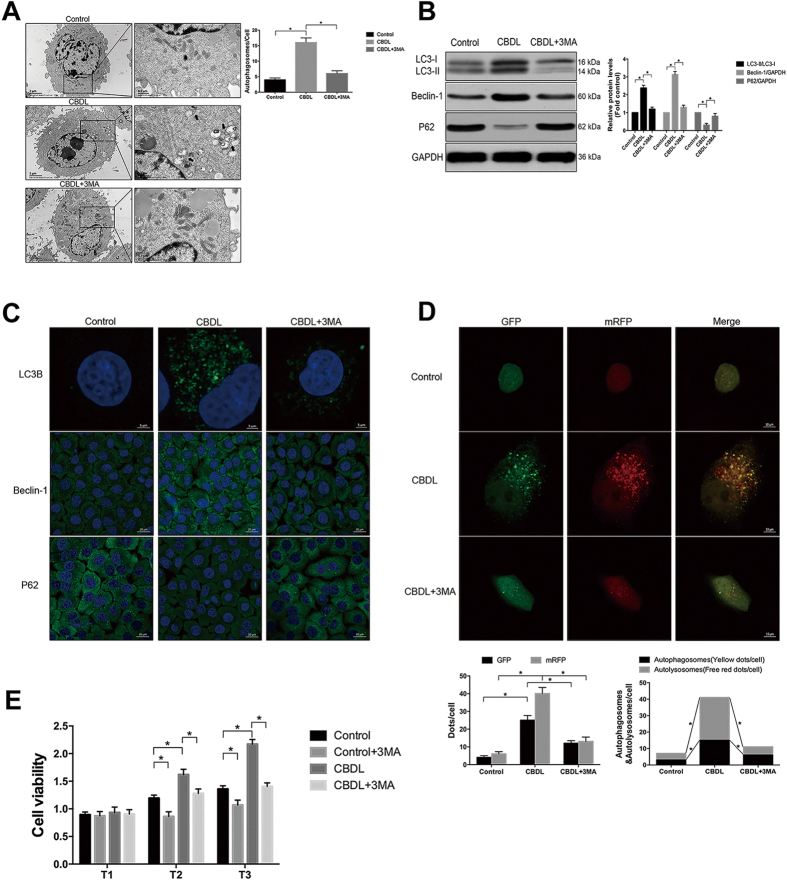
Inhibition of autophagy with 3MA (5 mM) attenuated the excessive proliferation of PMVECs induced by CBDL rat serum *in vitro*. (**A**) Representative TEM images and graphical summaries of autophagosomes in cultured PMVECs under different treatments in the absence or presence of 3MA. Broad arrows represent autophgosomes. Asterisks represent autolysosomes. 30 random cells for each group were observed. (**B**) Western blotting and graphical summaries of LC3B, Beclin-1 and p62 protein levels in cultured PMVECs under different treatments in the absence or presence of 3MA for 24 h. All blots were representative of three independent experiments. (**C**) Representative immunocytochemistry images of LC3B, Beclin-1 and p62 protein in cultured PMVECs under different treatments in the absence or presence of 3MA for 24 h. (**D**) Representative confocal microscope images and graphical summaries of LC3 in different groups of PMVECs infected with mRFP-GFP-LC3 adenovirus for 24 h. 30 random cells for each group were observed. (**E**) Cell viability in different groups of PMVECs was determined by CCK-8 analysis. Data were from three independent experiments. Values were expressed as means ± SEM. *P < 0.05.

Since the pathological process of HPS is highly related with the excessive proliferation of PMVECs, we asked whether the inhibition of autophagy activation might alleviate the induced proliferation of PMVECs. To determine the role of autophagy in CBDL rat serum-induced excessive proliferation of PMVECs, cultured PMVECs were treated with autophagy inhibitor 3-MA. 3MA(5 mM) treatment effectively blocked autophagy activation in cultured PMVECs at 24 h after CBDL rat serum stimulation, as evidenced by the inhibition of autophagosome formation, the reduced protein levels of LC3B and Beclin-1 as well as the increased protein levels of P62, and the repression of autophagic flux with the much lower amounts of autophgic structures (Fig. 4A–C). Next, CCK-8 analysis was applied in order to check the effect of autophagy inhibition on cell proliferation ability. CCK-8 analysis demonstrated that autophagy inhibition with 3MA treatment efficiently repressed CBDL rat serum-induced proliferation of PMVECs at each time-point (Fig. 4D). These findings suggest that autophagy activation may contribute to CBDL rat serum-induced excessive proliferation of PMVECs, and 3MA can attenuate this alteration through inhibiting the autophagy activation.

## Discussion

HPS, a defective liver-induced pulmonary vascular disorder, is characterized by worsening hypoxemia due to intrapulmonary vascular dilatation (IPVD), arteriovenous malformations and increased vasoactive substances in the setting of chronic liver disease (CLD)^17^,^25^. Over the past two decades, the pathogenesis and precise mechanisms of HPS were under active investigation. Although much progress has been made in delineating the mechanisms underlying the imbalance of vasoactive substances, pulmonary vascular alterations and angiogenesis in HPS, additional mechanisms may involve in this disease^26^,^27^,^28^,^29^. Recently, many studies have demonstrated that autophagy is involved in various diseases, especially lung diseases^30^,^31^,^32^,^33^. So it is interesting and important to check whether autophagy might be related to HPS and whether autophagy plays a critical role in HPS. Our present work is the first one to evaluate autophagy activation in the development of HPS.

Autophagy, a self-digestion and dynamic process, is involved in long-lived proteins and dysfunctional organelles degradation^34^,^35^. The basal level of autophagy is essential for homeostasis, and the altered autophagy has been demonstrated in various pathological alterations. Although the beneficial effects of autophagy after lung injury have been demonstrated by some studies, in some cases, pharmacological inhibition of autophagy after some insults has protective effects^16^,^36^,^37^,^38^.

Our *in vivo* data suggest that autophagy was over-activated at the early time point (2-week) after CBDL and has a slight recovery at the later time point (4-week). This is a time-dependent alteration in autophagy levels after CBDL, which indicates that autophagy may have a certain-limited time course in the development of HPS. We expect that the over-activated autophagy in the early time after CBDL contributes to the development of HPS. And, in the present study we found that 3-MA treatment from the 1^st^ day and end on the 2^nd^ weeks post CBDL significantly blocked autophagy activation and improved the pathological alterations *in vivo*, which further demonstrate this point. Therefore, we propose that an early intervention aimed at the reduction in autophagosomes accumulation has the protective effects after CBDL. However, it is worth noting that a prolonged treatment with 3-MA has been demonstrated to promote autophagy flux under nutrient-rich conditions due to its differential temporal effects on class I and class III PI3K^39^. Such mechanism gives us an insightful thinking that the effect of 2-week 3-MA administration may be different from that of 4-week. In addition, it is still unclear why the autophagy status has a slight recovery in the late stage of CBDL rats and whether this alteration plays a regulatory role in HPS. All these important and interesting sections mentioned above need further research.

Our *in vitro* data further demonstrate that the above protective effect may result from the inhibition of PMVECs excessive proliferation and pulmonary microvascular dilation. Takeshi *et al*. demonstrated that autophagy gets induced in ECs in response to the pro-apoptotic agent, sulforaphane, and the inhibition of autophagy potentiates the pro-apoptotic effect^40^. Their findings open premises for the use of autophagy inhibitors in combination with anti-angiogenic agents, and also enlighten us about that autophagy may play a regulatory role in PMVECs excessive proliferation during HPS progression. In the present study, we demonstrated, for the first time, that autophagy activation may contribute to CBDL rat serum-induced proliferation of PMVECs, and 3-MA attenuates this alteration through inhibiting the autophagy activation. This finding provides another important therapeutic strategy for HPS. The development of autophagy inhibitors with higher specificity for ECs as well as angiogenic endothelium is desired to be tested in clinical trials.

Based on the above findings, we have a deep thinking about why autophagy was activated after CBDL and how autophagy modulates PMVECs proliferation. Here, we give some speculation about these questions. In recent years, autophagy regulation is under active investigation. It is worth noting that some common stimuli for autophagy activation, such as hypoxia, ER stress and inflammatory mediators, are also involved in the pathogenesis of HPS^8^,^41^,^42^. Moreover, Li *et al*. demonstrated that in bacteria-induced lung injury, Annexin A2 induces autophagy activation through inhibiting Akt1-mTOR-ULK1/2 signaling pathway^43^. Coincidently, our previous research found that CBDL rat serum induces Annexin A2 expression, which further contributes to the HPS-associated angiogenesis through ERK1/2 and NF-kB signaling pathway^44^. So the above factors might be responsible for the autophagy activation after CBDL. About the modulation of PMVECs proliferation, the interplay of autophagy and apoptosis should be considered. Autophagy (‘self-eating’) and apoptosis (‘self-killing’) determine the turnover of cytoplasmic organelles and entire cells, respectively. Although both autophagy and apoptosis are under the control of multiple common upstream signals, these processes also cross-regulate each other, mostly in an inhibitory manner. As such mechanism, autophagy activation can reduce cell death through selectively inhibiting the abundance of pro-apoptotic proteins, which further promotes cell proliferation^45^. This is an interesting part and we need more support data to demonstrate it in the following research.

Another interesting question is whether the effect of autophagy inhibition comes from inhibition of intrapulmonary angiogenesis or is secondary to amelioration of cirrhosis. It is worth noting that *in vivo* although we found that 3-MA intervention alleviate the pulmonary pathological alterations, but the rats still presented obvious ascites which is an indicator of Child-Pugh score. We hypothesized that the effect of autophagy inhibition mainly comes from inhibition of intrapulmonary angiogenesis. However, this hypothesis needs more data to be supported. For example, we should use another administration method to demonstrate the targeted therapeutic effects, such as aerosol inhalation. And, we should also explore the impact on liver.

As we know, HPS is a really complex syndrome in which multiple factors change obviously. Recently, it is hard to answer which mechanism plays a dominant role in HPS. In the early phases of CBDL, increased bilirubin, endotoxin and inflammatory mediators not only cause the liver injury but also are released into the circulatory system to damage distant organs. These injuries further evoke the self-repair mechanisms, thus promoting the release of various growth factors and cytokines such as ET-1, TNF-α and VEGF-A. Theses molecules activate the survival signals, such as Akt and ERK, which may in turn lead to ECs proliferation and pathological alterations of HPS^24^. Compared with these factors mentioned above, autophagy seems to be their common downstream which is a cellular housekeeping process to remove damaged proteins and organelles through an alternative degradation mechanism (i.e., nonproteasomal) and serves as an adaptive response to maintain cell survival. Further research is needed to address mechanism difference attributed to autophagy and factors mentioned above.

**Figure 5:**
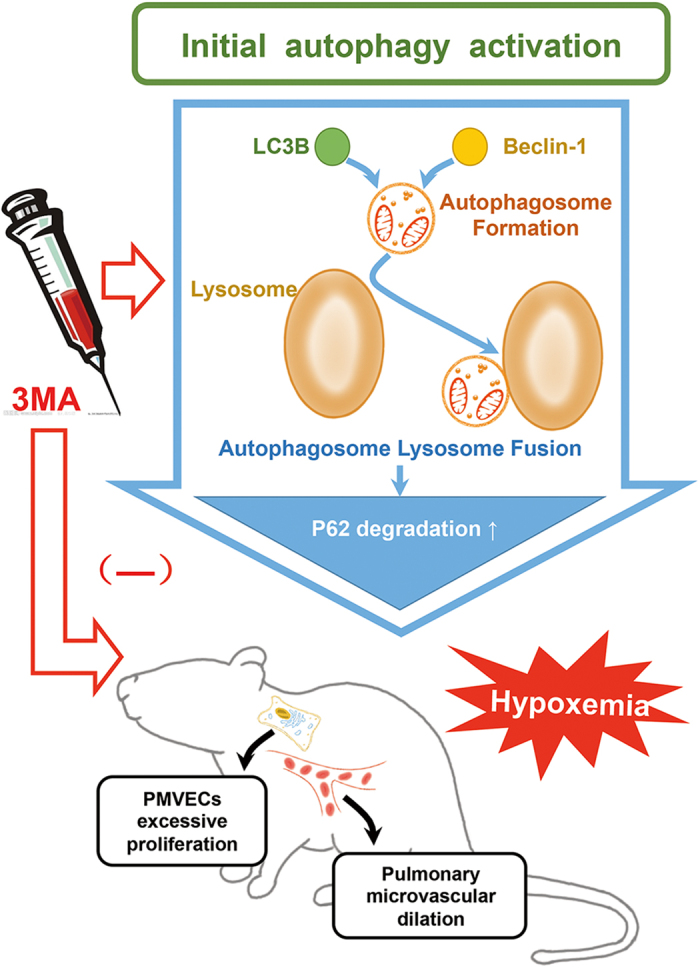
Model of the initial autophagy activation and beneficial effects of 3-methyladenine (3-MA) intervention in rat experimental hepatopulmonary syndrome. Pharmacological inhibition of autophagy with 3MA improved pathological alterations -*pulmonary microvascular dilation and PMVECs excessive proliferation* and typical symptom*-hypoxemia* in rat experimental hepatopulmonary syndrome.

In conclusion, our study provides new insights into the role of autophagy in the pathogenesis of HPS. From our study of *in vivo* animal model, we demonstrated that an early intervention aimed at the reduction in autophagosomes accumulation has the protective effects on HPS-associated pathological alterations and serious hypoxemia. From our study of *in vitro* cultured cell model, we further demonstrated that this protective effect might result from the inhibition of PMVECs excessive proliferation. We conclude that the initial overactive autophagy may at least partly contribute to the development of HPS in CBDL rats (Fig. 5). This finding may provide a new strategy for the clinical management of HPS or other proliferative vascular diseases.

## Materials and Methods

### Animal model

Male Sprague-Dawley rats (200–220 g, 6 weeks, Third Military Medical University, Chongqing, China) were used in this study. An experimental HPS rat model was successfully established by common bile duct ligation (CBDL) as previously described^46^,^47^. The experimental group underwent common bile duct ligation. The control group underwent common bile duct exposure but no ligation. The autophagy inhibitor 3-methyladenien (Sigma-Aldrich, St Louis, Missouri, USA) was administrated daily by intraperitoneal injection (15 mg/kg) within 2 weeks following CBDL. The vehicle group animals were injected with the same volume of 0.9% saline. Specimens were collected at the end of 2 and 4 weeks, respectively. All rats were housed under a standard diet and living conditions (22–24 °C,12 h light/12 h dark cycle). All procedures performed on the animals were conducted according to the guidelines from the National Institutes of Health. In addition, all experimental protocols were approved by the ethical committee of Third Military Medical University.

### Cell culture

Cultured rat PMVECs were isolated from healthy Sprague-Dawley rats as previously described^18^,^48^. Cells were cultured in endothelial cell medium (ECM) with 10% fetal bovine serum (FBS), 100 U/ml of penicillin-streptomycin and 1% endothelial cell growth supplement in a 95% O_2_/5% CO_2_ incubator at 37 °C.PMVECs were incubated with 10% normal rat serum or 10% CBDL rat serum for 0 h (T1), 12 h (T2) and 24 h (T3). Experimental data were obtained from cells between passages third to six.

### Transmission electron microscopy

Lung tissues and PMVECs were collected and fixed in 2% paraformaldehyde and 0.1% glutaraladehyde in 0.1 M sodium cacodylate for 2 h, post-fixed with 1% O_S_O4 for 1.5 h, washed, and stained for 1 h in 3% aqueous uranyl acetate. And then lung tissues and cells were washed again, dehydrated by graded alcohol and embedded in Epon-Araldite resin (Canemco & Marivac, Quebec, Canada). Ultrathin sections were cut by an ultra-microtome (Reichert-June, Inc., Cambridge, UK), counterstained with 0.3% lead citrate and observed under a transmission electron microscope (model: EM420; Koninklijke Philips Electronics N.V., Amsterdam, The Netherlands).

### Western blotting

The protein samples extracted from lung tissues of rats or cultured PMVECs were subjected to SDS-PAGE gels and transferred onto PVDF membranes (Millipore, Billerica, MA, USA). Membranes were blocked for 1 h using 5% skim milk in TBST at room temperature, and then incubated with appropriate primary antibodies overnight at 4 °C (LC3B:1:1000, Beclin-1:1:1000, p62:1:1000; Abcam, Cambridge, MA, USA), followed by incubation with secondary antibody (HRP-conjugated rabbit anti-goat IgG 1:10000; Abcam, Cambridge, MA, USA). Finally, the membranes were visualized using a gel imaging system (Bio-Rad Laboratories, Hercules, CA, USA). The optical density of immnoreactivity was analyzed with an Alpha Imager (Protein Simple, San Francisco, CA, USA).

### Immunofluorescence

PMVECs were fixed with 4% formaldehyde for 30 min, permeability with 0.3% Triton X-100 for 10 min and blocked with 10% goat serum for 1 h at room temperature. Cells were then incubated with appropriate primary antibodies overnight at 4 °C (LC3B:1:200, Beclin-1:1:200, p62:1:200; Abcam, Cambridge, MA, USA) followed by Alexa Fluor 488-labelled secondary antibody (Abcam, Cambridge, MA, USA). DAPI were used for nuclear staining (Beyotime Inc., Shanghai, China). Micrographs were obtained with a fluorescent microscope (Olympus BX51, Tokyo, Japan).

### Autophagy detection using mRFP-GFP-LC3 adenovirus

PMVECs were seeded onto cover slides and allowed to reach 50–70% confluence before transfection. Adenoviral infection was performed according to the manufacturer’s instructions. PMVECs were incubated in growth medium with mRFP-GFP-LC3 adenovirus (HanBio Technology Co., Shanghai, China) at 30 MOI. After 12 h, the transfected cells were exposed to various indicated treatments. Then cells were washed with PBS, fixed by 4% paraformaldehyde and analyzed by confocal microscope (Olympus, Tokyo, Japan).

### Arterial blood gas analysis

All animals were anaesthetized by intraperitoneal injection of sodium pentobarbitone (40 mg/kg). Arterial blood was drawn from the abdominal aorta and further analyzed using ABL 700 radiometer (Radiometer, Copenhagen, Denmark). The assessment of HPS in CBDL rats was based on the following criteria: gas exchange dysfunction (PaO_2_ < 85 mmHg, A-aDO_2_ > 18 mmHg)^49^. Serum was separated from blood samples (7–8 ml), and then centrifuged at 2000×g/min in a Gyria for 10 min at 4 °C. Following filtration with cellulose acetate membranes, serum was further inactivated at 56 °C for 30 min and stored at −80 °C for use in the subsequent experiments.

### Pulmonary wet-to-dry ratio

Pulmonary edema was assessed by pulmonary wet-to-dry ratio as previously described^50^. Briefly, lung tissues were weighed before and after storing at 80 °C for three days, and then the wet-to-dry ratio was calculated.

### Fluorescent-labeled microsphere assay

Fluorescent-labeled microspheres (Life Technologies, Carlsbad, CA, USA) in 0.2 ml of sterile distilled water were injected over 10 s through the jugular catheter, which was immediately flushed with 0.2 ml of sterile saline over 10 s. After 30 min injection, lung and brain samples were collected and homogenized. The fluorescent intensity was measured at 580/605 nm using a Multiscan Spectrum (Molecular Devices, Sunnyvale, CA, USA). And then, the ratio of brain/lung fluorescence intensity was calculated.

### Histological analysis

Lung tissues were collected and fixed for histological analysis as previously described^24^. Briefly, after lung tissues were fixed in 10% formalin for 48 h, dehydrated in alcohol, embedded in paraffin, cut into 5-um thickness sections and stained with haematoxylin and eosin (H&E). The microphotographs of the specimens were obtained with a light microscope (Olympus, Tokyo, Japan).

### CCK-8 assay

Cell proliferation was detected by the Cell Counting Kit-8 assay (Dojindo, Kumamoto, Japan). 24 h after the same number of PMVECs was seeded in 96-well plates (0.8−1.0 × 10^4^ cells per well), cells were pretreated with autophagy inhibitor 3MA (5 mM) prior to the addition of DMEM containing different sera for the indicated time. At the end of treatment, 10 ul CCK-8 solution was added to each well, and cells were cultured for 2 h at 37 °C. After that, viable cells were detected by measuring the absorbance value at 450 nm using a Multiscan Spectrum (Molecular Devices, Sunnyvale, CA, USA).

### Statistical analysis

All data were expressed as the mean ± SEM and analyzed using SPSS 17.0 statistical software (SPSS Inc., Chicago, IL, USA). Multiple comparisons between groups were analyzed with Bonferron-i-corrected analysis of variance (ANOVA), and the remaining data were analyzed with Student’s *t*-test. A *P* value <0.05 was considered statistically significant.

## Additional Information

**How to cite this article**: Xu, D. *et al*. Inhibition of autophagy ameliorates pulmonary microvascular dilation and PMVECs excessive proliferation in rat experimental hepatopulmonary syndrome. *Sci. Rep*. **6**, 30833; doi: 10.1038/srep30833 (2016).

## Figures and Tables

**Table 1:** Effect of the autophagy inhibitor 3-methyladenine (3MA) on arterial oxygenation in 2- and 4-week CBDL rats.

	Sham	CBDL		CBDL+3MA	
		2 weeks	4 weeks	2 weeks	4 weeks
*PO*_*2*_ (mmHg)	92 ± 2.1	75.9 ± 3.5*	71.8 ± 3.5*	80.3 ± 3.2#	82.7 ± 3.1##
*PCO*_*2*_ (mmHg)	42.3 ± 2.3	41.3 ± 3.1	42.5 ± 3.3	42.3 ± 2.1	41.9 ± 2.3
*A_a_PO*_*2*_ (mmHg)	6.9 ± 2.7	19.7 ± 2.9*	23.1 ± 2.7*	14.6 ± 3.7#	12.1 ± 3.2##

^*^P < 0.05 compared with sham.

^#^P < 0.05 compared with CBDL 2 weeks.

^##^P < 0.05 compared with CBDL4 weeks.

Values are expressed as means ± SEM (n = 8). CBDL, common bile duct ligation; *A*_a_*PO*_2_, alveolar-arterial oxygen gradient.
